# The Coddling of Children to Prevent Colds

**Published:** 1899-04

**Authors:** 


### THE CODDLING OF CHILDREN TO PREVENT COLDS.

It has been said that it is always a misfortune for parents to have only one child, since the care and solicitude over the general well-being of such causes a great deal of disquietude on the part of parents. Such a child is usually so extremely cared for that it suffers in consequence. Then, again, a great many parents think that whenever cold or bad weather comes the children must be kept indoors, or if permitted to go outside, they must be severely wrapped in outer-garments from head to foot, and large mufflers must be placed around the throat. This method of management is what is commonly called "coddling," i. e., bundling children up whenever they go outside, and not permitting them often even this privilege. We honestly believe that such treatment is productive of more colds, rendering such a person more liable to colds than all the exposure to the roughest weather. Of late it has been found that if children who are subject to croups and colds every winter be made to go barefooted all the year round, that such will prove a positive cure to this condition. Experience has demonstrated the truthfulness of this statement. Children should be raised, not in the house, but under the canopy of nature's covering. Just in this line a recent editorial in that excellent journal, *Pediatrics*, is well worthy of quotation. It says:

"The crusade now being undertaken against tuberculosis has clearly brought to the minds of every one that the only intelligent treatment of the disease is by means of fresh, pure air and a good diet. In all parts of the world, sanitariums, conducted on these principles, are springing up, and even in England, where one would think that the climate was hardly suitable to the out-of-door treatment, the system is to be carried out on a large scale. But, after all, it should be remembered if proper care is taken of children when young, and if their bringing-up is carried out more with the view of hardening them, and thus rendering them proof against the ubiquitous microbe, the need of sanitariums would be much less than is at present the case. It is a fact, both instructive and pertinent, that in many of the coldest portions of the globe colds are unknown. Nansen and his men, when in the Arctic regions, although they underwent exposure of every description, never once suffered from colds, but no sooner had they set their foot on their native shore of Norway than they, one and all, caught severe colds. The experience of other Arctic explorers is the same. It seems then probable that there may be something in the theory of the infectiousness of colds, and that we shall have to give up our traditional belief to the contrary, however much we may have treasured it. If the infection theory be the true one, and if it be frankly accepted, a radical change in the method of treating colds must necessarily follow. If exposure is not the direct cause, but merely acts by so lowering the vitality that the germs can gain an easy foothold, the rational treatment must be to build up and harden the constitution in such a manner that it will refuse to harbor the seeds of disease. Mothers and nurses are too much afraid of fresh air and ventilation, but when they understand that the hothouse plan of rearing children will produce a nation of weakly, delicate individuals, susceptible to every complaint that is about, they will doubtless see the error of their ways."

